# Supplementary material for: Deletion of wfs1 Impairs Oligodendrocyte Precursor Cells Dorsal Distribution and Myelination Through the wfs1-hmgcs1 Axis in Zebrafish
Source: Biology (Basel). 2026 Aug 16;15(16):1402. doi: 10.3390/biology15161402 (PMC13510151; doi:10.3390/biology15161402)
Supplement: Supplementary file 1 [file biology-15-01402-s001.zip › File S1-Transcriptome sequencing data and differentially expressed genes (DEGs) in wfs1-- zebrafish at 5 dpf/2.KEGG/wfs1KOvsWT/ALL/src/dre04350.html]

dre04350


- dre:323883

- Down regulated genes

323883(-4.8491500403135)

- dre:566841

- Up regulated genes

566841(1.91411798034227)

- dre:100004116

- Up regulated genes

100004116\_1(2.49931465780725)

- dre:436718

- Up regulated genes

436718(1.34229427553561)

- Down regulated genes

436718\_1(-6.85571323128554)

- dre:559723

- Up regulated genes

559723(3.63719210583924)

- dre:100332300

- Up regulated genes

405778(4.96269681782231)

- dre:556634

- Down regulated genes

556634(-1.23964511809438)

Close
